# *In silico* Design of Novel HIV-1 NNRTIs Based on Combined Modeling Studies of Dihydrofuro[3,4-d]pyrimidines

**DOI:** 10.3389/fchem.2020.00164

**Published:** 2020-03-24

**Authors:** Yanming Chen, Yafeng Tian, Ya Gao, Fengshou Wu, Xiaogang Luo, Xiulian Ju, Genyan Liu

**Affiliations:** ^1^Key Laboratory for Green Chemical Process of Ministry of Education, Hubei Key Laboratory of Novel Reactor and Green Chemical Technology, School of Chemical Engineering and Pharmacy, Wuhan Institute of Technology, Wuhan, China; ^2^School of Materials Science and Engineering, Zhengzhou University, Zhengzhou, China

**Keywords:** HIV-1 non-nucleoside reverse transcriptase inhibitors (NNRTIs), dihydrofuro[3, 4-d]pyrimidines, virtual screening, molecular docking, rational drug design

## Abstract

A novel series of dihydrofuro[3,4-d]pyrimidine (DHPY) analogs have recently been recognized as promising HIV-1 non-nucleoside reverse transcriptase (RT) inhibitors (NNRTIs) with potent antiviral activity. To better understand the pharmacological essentiality of these DHPYs and design novel NNRTI leads, in this work, a systematic *in silico* study was performed on 52 DHPYs using three-dimensional quantitative structure–activity relationship (3D-QSAR), molecular docking, virtual screening, absorption-distribution-metabolism-excretion (ADME) prediction, and molecular dynamics (MD) methods. The generated 3D-QSAR models exhibited satisfactory parameters of internal validation and well-externally predictive capacity, for instance, the q^2^, R^2^, and ${{\text{r}}_{\text{pred}}}^{2}$ of the optimal comparative molecular similarity indices analysis model were 0.647, 0.970, and 0.751, respectively. The docking results indicated that residues Lys101, Tyr181, Tyr188, Trp229, and Phe227 played important roles for the DHPY binding. Nine lead compounds were obtained by the virtual screening based on the docking and pharmacophore model, and three new compounds with higher docking scores and better ADME properties were subsequently designed based on the screening and 3D-QSAR results. The MD simulation studies further demonstrated that the newly designed compounds could stably bind with the HIV-1 RT. These hit compounds were supposed to be novel potential anti-HIV-1 inhibitors, and these findings could provide significant information for designing and developing novel HIV-1 NNRTIs.

## Introduction

Acquired immune deficiency syndrome (AIDS) caused by human immunodeficiency virus (HIV) is one of the most widely spread infectious diseases worldwide. There is no effective drug or vaccine that could cure AIDS absolutely at present. According to the report from the Joint United Nations Program on HIV/AIDS, there were approximately 36.9 million people living with HIV worldwide in 2018, and neighboring 1.8 million new cases and 0.94 million AIDS-related deaths in 2017^1^. Two main types of HIV (HIV-1 and HIV-2) have been identified currently. HIV-1 is widely spread throughout the world, whereas HIV-2 has correspondingly poor transmission (Vasavi et al., 2019; Wang et al., 2019). In the fight against HIV-1, highly active antiretroviral therapy (HAART) has been considered to be a relatively successful and effective therapy in controlling HIV-1 epidemics (Chen et al., 2011; Wang et al., 2018).

HIV-1 reverse transcriptase (RT), as one of the most important enzymes that convert the single-stranded RNAs into double-stranded DNAs, is vital to restrain HIV-1 replication and a prime target for antiviral research (Esposito et al., 2012). Inhibitors of the HIV-1 RT are divided into nucleoside RT inhibitors (NRTIs) and non-nucleoside RT inhibitors (NNRTIs), and the latter binds to an allosteric site that is located about 10 Å distance from the polymerizing processing site (Zhan et al., 2009). NNRTIs have become an indispensable portion of HAART regimen due to its potent antiviral activity, high specificity, and low cytotoxicity. However, single mutations such as K103N, Y181C, V106A, and L100I in the binding site of the HIV-1 RT might result in decreased inhibitory potencies of NNRTIs, and a double mutation (K103N+Y181C) was more frequently discovered in the process of treating with NNRTIs (Das et al., 2008).

**Figure 1:**
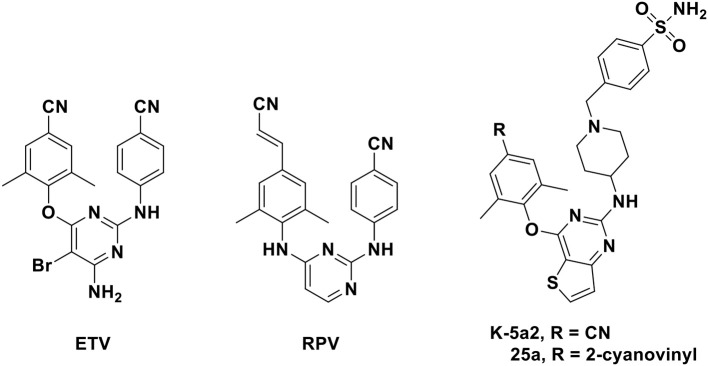
Chemical structures of diarylpyrimidines (DAPYs).

Six HIV-1 NNRTIs including nevirapine, delavirdine, efavirenz, etravirine (ETV), rilpivirine (RPV), and doravirine have been approved by US Food and Drug Administration for clinical use to date (Namasivayam et al., 2019). ETV and RPV (Figure 1), which belong to diarylpyrimidine (DAPY) derivatives that were recognized as one of the most effective families of NNRTIs, have attracted considerable attention due to their excellent potency against HIV-1 wild-type and mutant strains. However, the low solubility and unsatisfactory oral bioavailability of these analogs restrict their clinical usage in some respects (Gu et al., 2019). Thus, novel NNRTIs with improved pharmacokinetic profiles have been urged to design and discover.

Recently, Kang et al. (2016, 2017) have designed and synthesized a series of thiophene[3,2-d]pyrimidine derivatives, among which compounds **K-5a2** and **25a** (Figure 1) were two representative HIV-1 NNRTIs, exhibiting more drug-like pharmacokinetic properties and greater inhibitory activities compared to nevirapine and efavirenz. Compound **25a** also exhibited better inhibition against HIV-1 mutant strains than ETV and RPV. However, compound **K-5a2** did not display excellent activity against K103N+Y181C mutant HIV-1 strains (Kang et al., 2017; Yang et al., 2018). Further structural modification on **K-5a2** and **25a** using six alicyclic-fused pyrimidine rings led to a series of dihydrofuro[3,4-d]pyrimidine (DHPY) derivatives with potent anti-HIV activity (Table 1) (Kang et al., 2019).

**Table 1:** Chemical structures of DHPYs and their actual and predicted activities as HIV-1 NNRTIs.

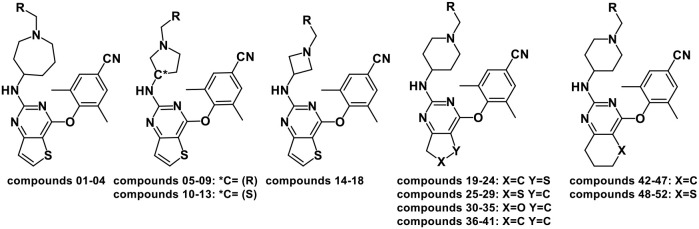							
				**CoMFA**		**CoMSIA**	
**No**.	***R***	**EC**_**50**_ **(nM)**	**Actual pEC**_**50**_	**Predicted pEC**_**50**_	**Residual**	**Predicted pEC**_**50**_	**Residual**
01^b^	4-SO_2_NH_2_-Ph	2.20	8.658	8.728	0.070	8.672	0.014
02	3-CONH_2_-Ph	10.3	7.987	8.043	0.056	8.014	0.027
03	4-SO_2_CH_3_-Ph	9.98	8.001	7.919	−0.082	7.992	−0.009
04^a^	4-pyridinyl	31.7	7.499	7.528	0.029	7.392	−0.107
05^b^	4-SO_2_NH_2_-Ph	8.69	8.061	8.046	−0.015	8.076	0.015
06	4-CONH_2_-Ph	10.4	7.983	8.042	0.059	8.025	0.042
07	3-CONH_2_-Ph	41.3	7.384	7.360	−0.024	7.365	−0.019
08	4-SO_2_CH_3_-Ph	13.9	7.857	7.923	0.066	7.860	0.003
09^a^	4-pyridinyl	16.0	7.796	7.500	−0.296	7.830	0.034
10	4-SO_2_NH_2_-Ph	104	6.983	7.083	0.100	7.004	0.021
11	4-CONH_2_-Ph	55.7	7.254	7.164	−0.090	7.242	−0.012
12	4-SO_2_CH_3_-Ph	50.7	7.295	7.214	−0.081	7.275	−0.020
13^b^	4-pyridinyl	16.7	7.777	7.843	0.066	7.781	0.004
14	4-SO_2_NH_2_-Ph	4.53	8.344	8.251	−0.093	8.335	−0.009
15	4-CONH_2_-Ph	4.76	8.322	8.317	−0.005	8.304	−0.018
16	3-CONH_2_-Ph	8.95	8.048	7.954	−0.094	8.053	0.005
17^a^	4-SO_2_CH_3_-Ph	207	6.684	6.860	0.176	7.329	0.645
18^b^	4-pyridinyl-Ph	2.21	8.656	8.690	0.034	8.680	0.024
19	4-SO_2_NH_2_-Ph	4.3	8.367	8.468	0.101	8.467	0.100
20^a^	4-CONH_2_-Ph	4.8	8.319	8.490	0.171	8.328	0.009
21	4-SO_2_CH_3_-Ph	5.9	8.229	8.197	−0.032	8.238	0.009
22^b^	4-pyridinyl	2.6	8.585	8.613	0.028	8.637	0.052
23^a^	4-NO_2_-Ph	8.0	8.097	8.103	0.006	8.226	0.129
24	3-CONH_2_-Ph	27.7	7.558	7.500	−0.058	7.547	−0.011
25^a^	4-SO_2_NH_2_-Ph	37.2	7.429	7.494	0.065	7.263	−0.166
26^a^^,^ ^b^	4-SO_2_Me-Ph	3.8	8.420	8.406	−0.014	7.714	−0.706
27	4-NO_2_-Ph	11.5	7.939	7.902	−0.037	7.898	−0.041
28	4-NH_2_-Ph	8.4	8.076	7.889	−0.187	8.067	−0.009
29	4-NHSO_2_Me-Ph	11.2	7.951	8.043	0.092	7.987	0.036
30	4-SO_2_NH_2_-Ph	2.8	8.553	8.562	0.009	8.543	−0.010
31^b^	4-CONH_2_-Ph	1.6	8.796	8.768	−0.028	8.812	0.016
32	4-SO_2_CH_3_-Ph	1.9	8.721	8.677	−0.044	8.629	−0.092
33	4-pyridinyl	2.3	8.638	8.701	0.063	8.736	0.098
34	4-NO_2_-Ph	7.4	8.131	8.075	−0.056	8.178	0.047
35^a^	3-CONH_2_-Ph	7.8	8.108	7.971	−0.137	8.238	0.130
36^b^	4-SO_2_NH_2_-Ph	1.1	8.959	8.867	−0.092	8.941	−0.018
37	4-CONH_2_-Ph	6.1	8.215	8.387	0.172	8.270	0.055
38^a^	4-SO_2_CH_3_-Ph	4.9	8.310	8.603	0.293	8.479	0.169
39	4-pyridinyl	1.8	8.745	8.642	−0.103	8.452	−0.293
40	4-NO_2_-Ph	14.5	7.839	7.953	0.114	7.858	0.019
41^a^	3-CONH_2_-Ph	2.0	8.699	8.060	−0.639	8.437	−0.262
42	4-SO_2_NH_2_-Ph	6.0	8.222	8.145	−0.077	8.169	−0.053
43^b^	4-CONH_2_-Ph	6.0	8.222	8.182	−0.040	8.218	−0.004
44^a^	4-SO_2_CH_3_-Ph	8.0	8.097	8.278	0.181	8.308	0.211
45	4-pyridinyl	8.6	8.066	8.033	−0.033	8.188	0.122
46	4-NO_2_-Ph	77.4	7.111	7.330	0.219	7.106	−0.005
47^a^	3-CONH_2_-Ph	6.5	8.187	7.792	−0.395	8.369	0.182
48^b^	4-SO_2_NH_2_-Ph	2.7	8.569	8.588	0.019	8.540	−0.029
49	4-CONH_2_-Ph	3.0	8.523	8.523	0.000	8.434	−0.089
50	4-SO_2_CH_3_-Ph	3.9	8.409	8.453	0.044	8.451	0.042
51	4-NO_2_-Ph	8.6	8.066	8.024	−0.042	8.056	−0.010
52^a^	3-CONH_2_-Ph	5.1	8.292	8.087	−0.205	8.429	0.137

a *Test set compounds used for 3D-QSAR models*.

b *The compounds used for pharmacophore models*.

*CoMFA, comparative molecular field analysis; CoMSIA, comparative molecular similarity indices analysis; DHPY, dihydrofuro[3,4-d]pyrimidine; NNRTIs, non-nucleoside reverse transcriptase inhibitors*.

To date, there are many computer-aided drug design methods applied in designing and developing novel HIV-1 inhibitors (Almerico et al., 2007). For example, the three-dimensional quantitative structure–activity relationship (3D-QSAR) and pharmacophore models were utilized to learn about structural characteristics of HIV-1 NNRTIs in our previous studies (Liu et al., 2018; Wan et al., 2018). The multivariate statistical procedures, containing principal component and discriminant analysis, could be as credible methods to predict the activities of HIV-1 inhibitors by taking advantage of the vast anti-HIV data (Almerico et al., 2003, 2006). The molecular docking and molecular dynamics (MD) simulation were often used to understand the binding conformations of ligands in the active sites of HIV-1-related proteins. Furthermore, a comparative analysis with the combination of docking and multivariate methods was used to study the drug resistance of HIV-1 inhibitors and to further design new compounds with appropriate structural features (Almerico et al., 2008).

To further explore the essential structural and pharmacological features of the novel DHPYs as HIV-1 NNRTIs in this study, the combination of 3D-QSAR models, molecular docking, and MD simulation was applied to analyze the 3D-QSARs of these DHPYs and their binding modes in the HIV-1 RT. We also utilized the pharmacophore- and docking-based virtual screening to obtain some hit compounds from ZINC database and subsequently designed new potential NNRTIs according to the screening and 3D-QSAR results. Molecular docking and MD simulations were utilized to identify the binding of these new NNRTIs and the stabilization of the protein–ligand complexes.

## Materials and Methods

### Preparation of Small Molecules

A total of 52 DHPY derivatives were collected from the published literature (Kang et al., 2019) for performing the molecular modeling study. Their structures, EC_50_, and corresponding pEC_50_ (−log*EC*_50_) values were listed in Table 1. All compounds were stretched by SYBYL-X 2.1 (Tripos Inc., St. Louis, USA) running on Windows 7 workstation and minimized with Gasteiger–Hückel charges, the termination of 0.005 kcal/(mol·Å) and max iterations of 1,000 by Powell method. Other parameters were set to default values.

### Three-Dimensional Quantitative Structure–Activity Relationship Model

**Figure 2:**
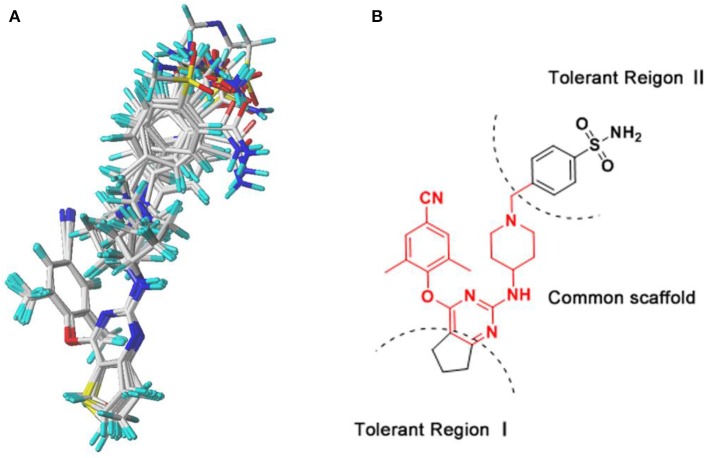
The molecular alignment using compound **36** as a template. **(A)** The alignment results of the training set compounds used for the three-dimensional quantitative structure–activity relationship (3D-QSAR) models. **(B)** The chemical structure of compound **36**, and the red region represents the common scaffold.

The 3D-QSAR model could help to find a significant correlation between the biological activities of drug molecules and their structures (Borisa and Bhatt, 2015). In this study, comparative molecular field analysis (CoMFA) and comparative molecular similarity indices analysis (CoMSIA) methods were used to construct 3D-QSAR models. All compounds were randomly divided into a training set (39 compounds) to generate CoMFA and CoMSIA models and a test set (13 compounds) to confirm the reliability of the generated models (Table 1). The number of test set compounds should be kept in the range from 1/4 to 1/3 of the total compounds. Compound **36** with the highest activity was used as a template, and all training set compounds were superimposed on it by the common skeleton alignment (Figure 2A).

For generating a reasonable model, the internal predictive ability was evaluated by partial least squares (PLS) regression method using the SAMPLS. The leave-one-out (LOO) cross-validation procedure was applied to determine the optimum number of components (ONC) and the highest cross-validation correlation coefficient (Q^2^) (Bush and Nachbar, 1993), and non-cross-validated analysis was applied to compute the non-cross-validated correlation coefficient (*R*^2^), standard error of estimate (SEE), and the Fisher test values (F) (Li et al., 2014). External validation parameters were also essential for further assessing the predictive capability of 3D-QSAR models, such as ${r_{0}}^{2}$, *k*, ${r_{0}}^{{}^{\prime }2}$, and *k*′. ${r_{0}}^{2}$, and *k* were the corresponding correlation coefficient and the slope value of linear regression equation, respectively, for predicted vs. actual activities when the intercept was set to zero, and ${r_{0}}^{{}^{\prime }2}$ and *k*′ were for actual vs. predicted activities, respectively. In addition, ${r_{m}}^{2}$, ${r_{m}}^{{}^{\prime }2}$, ${{△r}_{m}}^{2}$, $\bar{{r_{m}}^{2}}$, and the root mean square error (RMSE) as traditional data were also calculated to appraise the predictive ability. A model, which met the requirements of $\left[\left(r^{2}-{r_{0}}^{2}\right)/r^{2}\right]$ or $\left[\left(r^{2}-{r_{0}}^{{}^{\prime }2}\right)/r^{2}\right]$ < 0.1, 0.85 ≤ *k* ≤ 1.15 or 0.85 ≤ *k*′ ≤ 1.15, ${{△r}_{m}}^{2}$ < 0.2 and $\bar{{r_{m}}^{2}}$ > 0.5, especially the predictive correlation ${r_{pred}}^{2}$ > 0.6, would be deemed to possess well-predictive capability and reliability (Caballero, 2010; Ojha et al., 2011; Roy et al., 2016). The parameters were calculated according to our previous studies (Wang et al., 2018; Gao et al., 2019; Liu et al., 2019).

### Pharmacophore Model

Ten compounds (Table 1) with high activities and diverse structures were selected to generate pharmacophore model using Genetic Algorithm with Linear Assignment of Hypermolecular Alignment of Database (GALAHAD) module in SYBYL-X 2.1. GALAHAD method mainly contained two steps. The ligands are neatly aligned to each other in internal coordinate space, and then the produced conformations as rigid bodies are aligned in Cartesian space. In the process of running GALAHAD, the parameters of population size, max generation, and molecules required to hit were automatically set according to the experiment activity data. Finally, 20 models with diverse parameters including SPECIFICITY, N_HITS, STERICS, HBOND, and Mol_Qry were generated.

In order to further validate the ability of the pharmacophore model, a decoy set method was used for evaluating the generated model. The decoy set database was comprised of 6,234 inactive compounds downloaded from the DUD-E database (http://dud.docking.org/) (Mysinger et al., 2012) and 42 active compounds from Table 1 except the compounds used for constructing the pharmacophore model. The enrichment factor (EF) and Güner–Henry (GH) scores were considered as metrics to assess the reliability of the pharmacophore models. The GH score took the percent yield of actives in a hit list (%Y, recall) and the percent ratio of actives in a database (%A, precision) into account. While the GH score is ranging 0.6–1, the pharmacophore model would be regarded as a rational model (Kalva et al., 2014).

(1) $$\begin{array}{r} \%Y=H_{a}/H_{t}\times 100\% \end{array}$$

(2) $$\begin{array}{r} \%A=H_{a}/A\;\times 100\% \end{array}$$

(3) $$\begin{array}{r} EF=\left(H_{a}/H_{t}\right)/\left(A/D\right) \end{array}$$

(4) $$\begin{array}{r} GH=\left(H_{a}\;\left(3A+H_{t}\right)\right)/\left(4AH_{t}\right)\times \left(1-\left(H_{t}-H_{a}\right)/\left(D-A\right)\right) \end{array}$$

where H_a_ is the number of active molecules in the hit list, H_t_ is the hit compounds from the decoy set database, A is the total number of active compounds in the database, and D is the sum of the database.

### Molecular Docking

The crystal structures of wild-type HIV-1 RT (PDB ID: 6C0J) and K103N/Y181C mutant RT (PDB ID: 6C0R) were downloaded from the Protein Data Bank and were used for the docking study. While preparing the two proteins, hydrogen atoms were added after the crystallographic ligands were extracted and all water molecules except for W936 were removed. In order to verify the rationality and reliability of the docking method, the extracted ligands (**K-5a2** and **25a**) were first redocked into the corresponding active site using the Surflex-Dock Geom module of SYBYL-X 2.1 with default parameters. All compounds were then docked into the binding pocket as the same pattern. Twenty conformations with different scores were produced for each docked compound, and the highest-score conformation of each compound was chosen for further study.

### Virtual Screening

The selected GALAHAD model was converted into a UNITY query for virtual screening from ZINC database, and the “Flex search” was employed to serve as query type. Lipinski's rule of five as the primary filter was utilized to further decrease screened compounds. The QFIT score, whose value was between 0 and 100, reflected how closely the hit compounds matched with query. In consideration of the time and accuracy of screening, two ways of molecular docking including Surflex-Dock and Surflex-Dock Geom were implemented to verify the potential hit compounds obtained from the pharmacophore-based screening.

### ADME Analysis

ADME properties are essential for selecting and evaluating lead candidates. The online tool Swiss ADME (http://www.swissadme.ch/index.php) was applied to calculate the pharmacokinetic properties of new NNRTI candidates, such as lipophilicity, water solubility, and blood–brain barrier (BBB) permeability (Daina et al., 2017). The synthetic accessibility was also predicted by the score from 1 to 10, in which a score of 1 suggested the synthetic route was relatively easy and a score closer to 10 indicated the compound had complex structure and was tough to be synthesized.

### Molecular Dynamics Simulation

To further explore the dynamics protein–ligand interactions, 10 ns MD simulations were performed on compound **36** and newly designed inhibitors using GROMACS2016.5 software with AMBER 99SB force field. Before starting MD simulation, several important procedures should be performed to generate a steady environment. First, it was very momentous to generate the topology file of ligand by a acpype tool, which was on the basis of Python. Second, a 12 Å × 12 Å × 12 Å cubic box full of water models (transferable intermolecular potential with 3 points) was set to create the aqueous environment for the whole system. Nine chloride ions were added into the box for the sake of keeping the state of charge neutrality. In order to satisfy a tolerance of 10 kJ/mol, the steepest descent method for 50,000 steps was carried out for minimization without constraint to avoid possible collision between atoms. NVT at 300 K using V-rescale for 100 ps and NPT at 1 atm pressure using Parrine–Rahman for 100 ps were successively equilibrated to maintain proper temperature and pressure for the system. At last, the 10 ns MD simulation was run and the simulation step length was defined as 2 fs.

## Results and Discussion

### Statistical Analysis of the Comparative Molecular Field Analysis and Comparative Molecular Similarity Indices Analysis Models

The classical parameters of the CoMFA and CoMSIA models were summarized in Table S1. In general, the q^2^ and *R*^2^ should be more than 0.5 and 0.9, respectively, and the *SEE* and *F*-value should be rational. As for the CoMSIA models, there were different combinations of five fields as shown in Table S1. The model generated by the combination of the steric (S), electrostatic (E), hydrogen-bond acceptor (A), hydrogen-bond donor (D), and hydrophobic (H) fields was the optimal CoMSIA model because of its satisfactory q^2^, R^2^, *SEE*, F, and ${{\text{r}}_{\text{pred}}}^{2}$ values. The contributions of S, E, A, D, and H fields were 4.1, 19.7, 29, 33.4, and 13.8%, respectively, indicating that A and D fields played more important roles. The q^2^ of the CoMFA and CoMSIA models were 0.647 and 0.735, respectively, which indicated that both models were rational. The *R*^2^ values of the CoMFA and CoMSIA models were 0.970 and 0.982, respectively, and the ${{\text{r}}_{\text{pred}}}^{2}$ values were 0.751 and 0.672, respectively, suggesting that both models had excellent predictive abilities. In addition, it was common for the CoMFA and CoMSIA models that the E field contribution was more than the S field contribution, which illustrated that the E field could be more significant than the S field in the effect on compound activity.

**Figure 3:**
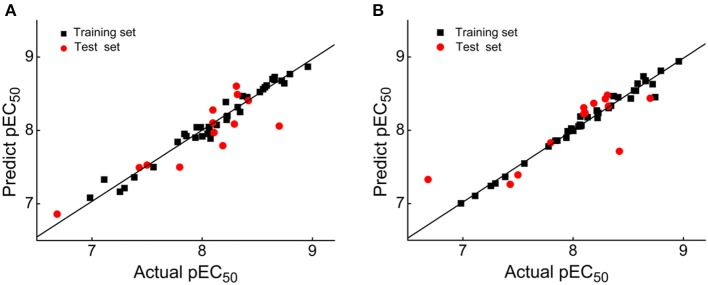
Plots of actual vs. predicted pEC_50_ values of all dihydrofuro[3,4-d]pyrimidines (DHPYs) based on the comparative molecular field analysis (CoMFA) **(A)** and comparative molecular similarity indices analysis (CoMSIA) **(B)** models.

External validation parameters could further confirm the reasonability of the constructed CoMFA and CoMSIA models. As shown in Table 2, all external validation results of the CoMFA and CoMSIA models were in the rational range, for example, the $\bar{{r_{m}}^{2}}$ values of the CoMFA and CoMSIA model were 0.648 and 0.524, respectively. The statistical results of Table S1 and Table 2 proved that the generated 3D-QSAR models were reliable and possessed excellent predictive capacity. Figure 3 showed the plots of actual vs. predicted pEC_50_ values for all compounds based on the CoMFA and CoMSIA models. All compounds were evenly distributed in the two sides of the trend lines, which indicated that the 3D-QSAR models had excellent abilities to predict the activities of DHPYs. The predictive capacity of the CoMFA model seems to be better than that of the CoMSIA model.

**Table 2:** External validation results of the CoMFA and CoMSIA models.

**Validation parameters**	**RMSE**	**MAE**	**r^2^**	**${{\text{r}}_{0}}^{2}$**	**r_0_^′2^**	**$\frac{{\text{r}}^{2}-{\text{r}}_{0}{}^{2}}{{\text{r}}^{2}}$**	**k**	**k^′^**	**${{\text{r}}_{\text{m}}}^{2}$**	**r_m_^′2^**	**${{△\text{r}}_{\text{m}}}^{2}$**	**$\bar{{{\text{r}}_{\text{m}}}^{2}}$**
CoMFA	0.263	1.608	0.750	0.746	0.709	0.006	1.007	0.992	0.700	0.597	0.103	0.648
CoMSIA	0.302	0.549	0.655	0.653	0.533	0.004	0.996	1.003	0.622	0.426	0.196	0.524

*CoMFA, comparative molecular field analysis; CoMSIA, comparative molecular similarity indices analysis; RMSE, root mean square error; MAE, mean absolute error*.

### Contour Maps of the Comparative Molecular Field Analysis and Comparative Molecular Similarity Indices Analysis Models

The contour maps of the CoMFA and CoMSIA models could visually provide significant information for the QSARs of DHPYs. Compound **36** with the highest activity was utilized as a reference molecule to analyze the contour maps of both models. As shown in Figure 2B, the structure of compound **36** consisted of the common scaffold, Tolerant Regions I and II.

**Figure 4:**
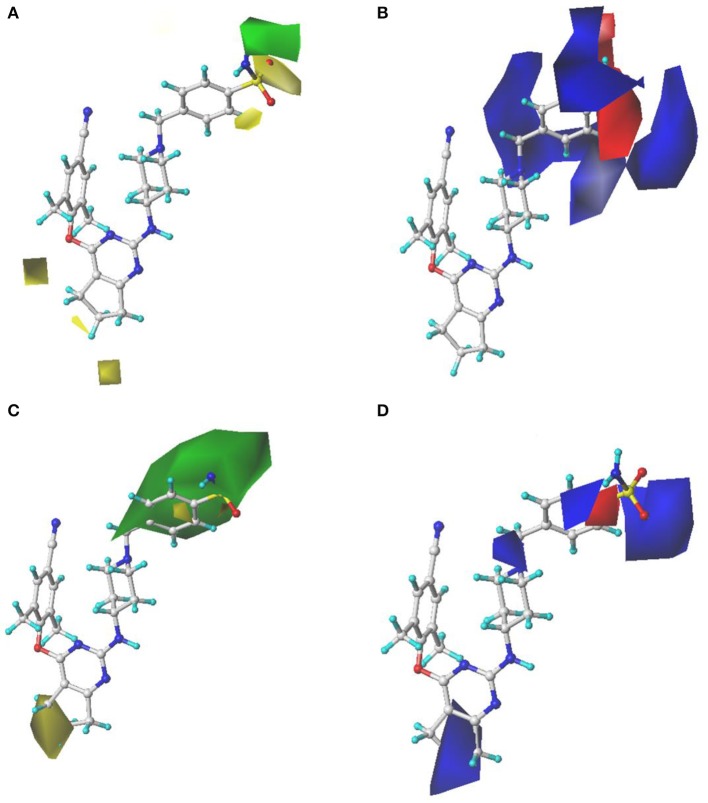
Contour maps of steric and electrostatic fields with compound **36** as a reference in the comparative molecular field analysis (CoMFA) **(A,B)** and comparative molecular similarity indices analysis (CoMSIA) **(C,D)** models.

Figure 4 showed the S and E field contour maps of the CoMFA and CoMSIA models. In the S field, the green contour indicates that a bulky substituent at this position is beneficial for the activity, whereas a yellow block corresponds to a region where a small group is favorable for the activity. For the E field, a blue contour means that introduction of electropositive groups in this region may improve the biological activity, whereas the red contour indicates that electronegative groups may be beneficial for the activity. As can be seen from Figures 4A,C, the S field contours of the CoMFA model were consistent with those of the CoMSIA model. The yellow contour in the Tolerant Region I indicated that a relatively small group at this region would be beneficial for enhancing the activity, which might explain why the actual activities of compounds **30**–**41** were greater than those of compounds **25**–**29**. On the other hand, in the Tolerant Region II, there was a green contour at the terminal, suggesting that introduction of a bulky group was more favorable, which was in agreement with the activity orders: **18** (pyridine-4-yl-Ph) > **14** (4-SO_2_NH_2_-Ph) > **17** (4-SO_2_CH_3_-Ph), **19** (4-SO_2_NH_2_-Ph) > **21** (4-SO_2_CH_3_-Ph) > **23** (4-NO_2_-Ph), and **42** (4-SO_2_NH_2_-Ph) > **44** (4-SO_2_CH_3_-Ph) > **46** (4-NO_2_-Ph). At the *para-position* of the benzene ring of Tolerant Region II, two yellow contours indicated that small substituents here might be favorable for the activity, for instance, **3** (4-SO_2_CH_3_-Ph) > **2** (3-CONH_2_-Ph) > **4** (pyridine-4-yl), **8** (4-SO_2_CH_3_-Ph) > **9** (pyridine-4-yl), **31** (4-CONH_2_-Ph) > **33** (pyridine-4-yl). In Figures 4B,D, it can be clearly observed that a big blue contour was located at the terminal of Tolerant Region II, indicating that the positively charged group might be beneficial for the activity, such as **1** (4-SO_2_NH_2_-Ph) > **3** (4-SO_2_CH_3_-Ph), **15** (4-CONH_2_-Ph) > **17** (4-SO_2_CH_3_-Ph), and **19** (4-SO_2_NH_2_-Ph) > **21** (4-SO_2_CH_3_-Ph). In addition, a red contour was located at the *para-position* of the benzene ring of Tolerant Region II, indicating that electronegative groups were beneficial for the antiviral activity at this position.

**Figure 5:**
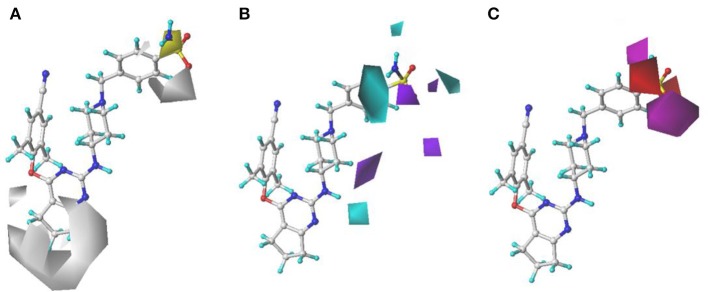
Contour maps of hydrophobic **(A)**, hydrogen-bond donor **(B)**, and hydrogen-bond acceptor **(C)** fields in the optimal comparative molecular similarity indices analysis (CoMSIA) model.

The H, D, and A field contour maps of the CoMSIA models were shown in Figure 5. In the H field, yellow contours represent the favorable zone of hydrophobic groups, whereas white contours show the unfavorable zone of hydrophobic groups. As shown in Figure 5A, a huge white near Tolerant Region I indicated that this place was appropriate to introduce hydrophobic groups. In addition, there was a white contour at the benzene ring of Tolerant Region II, which illustrated that hydrophobic substituents here were beneficial. The H field results were in good consistency with those of the previous study (Kang et al., 2019) that DHPYs with hydrophobic groups at corresponding positions exhibited promising activities. As for the D field, cyan suggests hydrogen-bond donor groups are useful for enhancing the activity, whereas purple is opposite. In Figure 5B, a cyan contour close to the linker atom of the pyrimidine ring and the right wing showed that the hydrogen-bond donor might be helpful for the activity at this position. There was also a cyan contour at the terminal of Tolerant Region II, indicating that hydrogen-bond donor groups were beneficial here, for example, **28** (4-NH_2_-Ph) > **27** (4-NO_2_-Ph). A purple contour near the para-position of the benzene ring of Tolerant Region II manifested that the place might not be suitable for hydrogen-bond donor groups, such as **1** (4-SO_2_NH_2_-Ph) > **2** (3-CONH_2_-Ph). In the A field, beneficial and unbeneficial contour of hydrogen-bond acceptors are colored in magenta and red, respectively. In Figure 5C, a red contour at the terminal of Tolerant Region II signified that the hydrogen-bond acceptors at this position were disadvantageous for the activity, and two magenta contours at the para-position of the benzene ring of Tolerant Region II illustrated that the hydrogen-bond acceptor was advantageous. In short, introduction of hydrogen-bond acceptors at the para-position of the benzene ring of Tolerant Region II and hydrogen-bond donors at the terminal of Tolerant Region II might be advantageous for the inhibitory activity.

In a word, the contour maps of 3D-QSAR models presented that a small and/or hydrophobic group in Tolerant Region I; a small, electronegative and/or hydrogen-bond accepter group at the *para-position* of the benzene ring of Tolerant Region II; and/or a bulky, electropositive and/or hydrogen-bond donor group at the terminal of Tolerant Region II would be favorable for increasing the activity, respectively.

### Pharmacophore Model

The statistical parameters of 20 pharmacophore models generated by GALAHAD were listed in Table S2. As for pharmacophore models, it could be served as the query for a UNITY flex search only if the SPECIFITY value was more than 5. The identical value of the PARETO column indicated that all models were statistically equivalent. In general, a good pharmacophore model should have small ENERGY and high SPECIFITY, N_HITS, STERICS, and MOL_QRY (Caballero, 2010). Among 20 models, model_20 was regarded as the optimal model by the comprehensive consideration of the abovementioned parameters.

**Figure 6:**
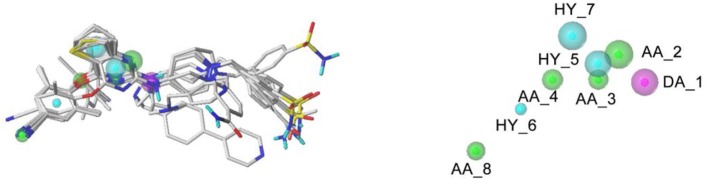
The best pharmacophore model with the alignment of 10 training set compounds. The model includes four hydrogen-bond acceptor atoms (green), three hydrophobic centers (cyan), and one hydrogen-bond donor atom (magenta).

The pharmacophore features of the best GALAHAD Model_20 were displayed in Figure 6, including three hydrophobic centers (HYs, cyan), four hydrogen-bond acceptor atoms (AAs, green), and one hydrogen-bond donor atom (DAs, magenta). All features were located in the left and middle structures of DHPYs. One of the hydrogen-bond acceptor atom at the connecting atom of the left ring indicated that hydrogen-bond acceptor groups might increase the inhibitory activities at this position, which was consistent with our previous study (Wan et al., 2018). The other hydrogen-bond acceptor atoms were located at the nitrogen atoms of the pyrimidine ring and the cyano group of the left benzene ring. Moreover, the hydrophobic center of the left phenyl ring was located at the hydrophobic pocket of the HIV-1 RT, which was also in good consistency with our previous studies (Wan et al., 2018). The right linker atom was the hydrogen-bond donor atom, which suggested that the hydrogen-bond donor atom at this position was likely to improve the anti-HIV-1 activities, which was in good agreement with the 3D-QSAR results.

For the optimal pharmacophore, there were 70 compounds screened from the decoy database, and 42 of them were active molecules. In addition, the calculated values of %Y, %A, EF, and GH were 60%, 100%, 89.66, and 0.70, respectively, which met the requirements that the EF value should be more than 1 and the GH value should be in the range from 0.6 and 1. These statistical results indicated that model_20 had excellent abilities of recognizing the false positives and distinguishing the similar structures of active and inactive compounds from the database. Thus, model_20 could be used for the next virtual screening studies.

### Molecular Docking

**Figure 7:**
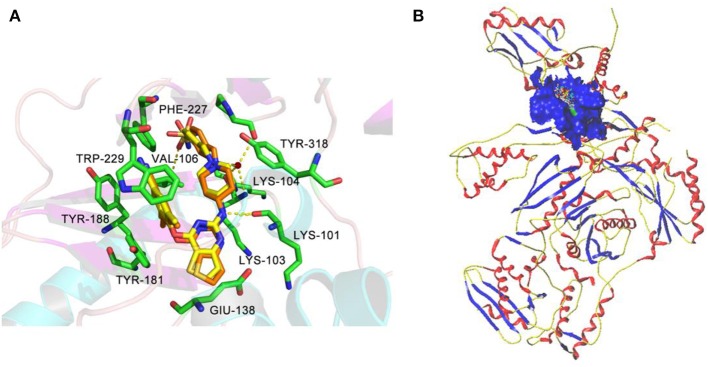
The redocked results of **K-5a2** in the binding pocket of the HIV-1 reverse transcriptase (RT) (PDB: 6C0J). **(A)** The superimposition of the cognate **K-5a2** (yellow) and the redocked **K-5a2** (orange). **(B)** The blue region represents the surface of the binding pocket.

Molecular docking was performed to investigate the binding modes of DHPYs at the active site of the HIV-1 RT. To validate the reliability of the molecular docking method, the cognate ligand (**K-5a2**) was redocked into the binding pocket of the HIV-1 RT (PDB: 6C0J), and the result was shown in Figure 7A. The original crystallographic and redocked conformations were almost superposition, and the root mean square deviation (RMSD) value between them for all atoms was 0.38 Å, which suggested that the docking method and used parameters were reasonable (Khan et al., 2010). As seen from Figure 7A, the two ligands adopted a similar binding pattern, in which the left benzene ring was located at the hydrophobic region consisting of residues Tyr181, Tyr188, Trp229, Phe227, and Val106 and could form π-π stacking interactions with the aromatic residues of them. In addition, it was noteworthy that the two ligands not only formed hydrogen-bond interactions with residues Lys101, Lys104, and Val106, respectively, but also interacted with residues Lys103 and Pro236 *via* a network of hydrogen bonds by a water molecule (W936). Those results were in good consistency with previous reports (Yang et al., 2018; Kang et al., 2019). At the same time, the hydrogen bond formed between the C = O of Lys101 and the NH of the right linker atom indicated that hydrogen-bond donor atoms were beneficial in the place, which was a good agreement with the results of pharmacophore and 3D-QSAR models. As shown in Figure 7B, 52 DHPYs embedded in the binging pocket by the similar *U*-shaped conformations, suggesting the accuracy of the docking method.

### Virtual Screening

**Figure 8:**
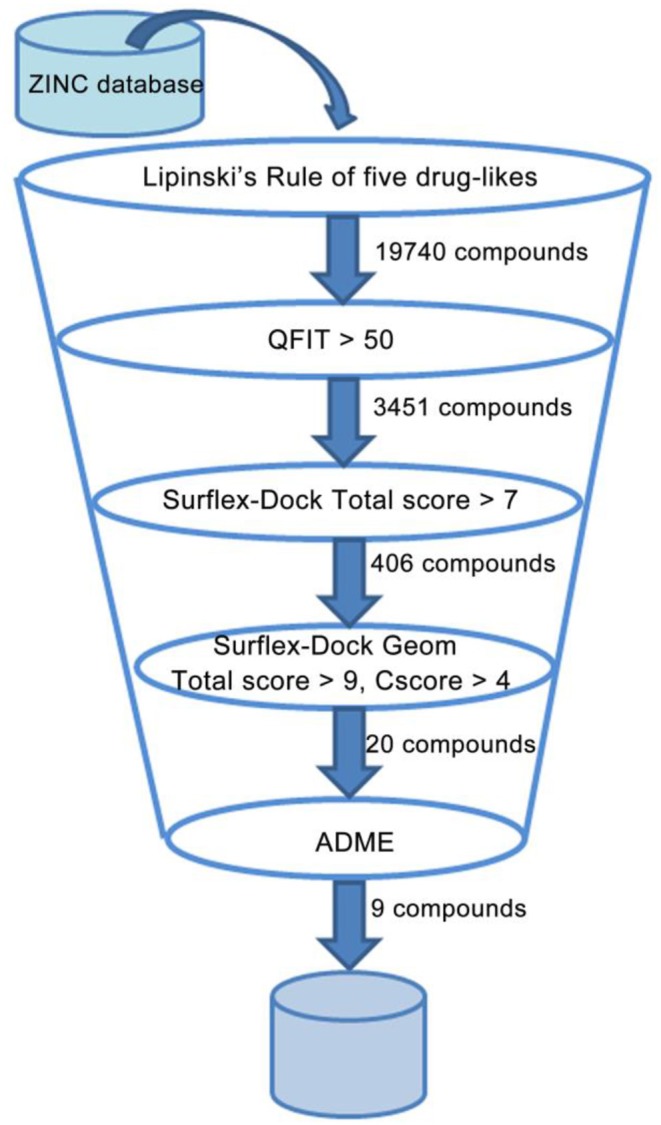
Representation of the overall virtual screening process.

To discover and design novel HIV-1 NNRTI leads, a multistage-filtered virtual screening was performed based on the constructed pharmacophore model and the established molecular docking method (Figure 8). First, a total of 19,740 compounds were obtained from ZINC database by the pharmacophore-based virtual screening and the restriction with Lipinski's rule of five. Then, 3,451 compounds were selected on the basis of the QFIT score of more than 50. In order to enhance the efficiency and accuracy of docking screening, the preliminary docking by Surflex-Dock and the second round docking by Surflex-Dock Geom were performed. The results indicated that only 20 compounds met the requirements simultaneously. In view of the predicted ADME properties of the screened 20 compounds, nine compounds were selected to regard as NNRTI hits, whose structures and docking scores were shown in Table 3. Furthermore, the interactions between the screened compounds and the HIV-1 RT were shown in Table S3. Nine screened compounds formed hydrophobic interactions with residues Tyr181, Tyr188, Phe227, Trp229, and Val106 and π-π stacking interactions with the aromatic residues of them. Except for ZINC_91409938, which formed a hydrogen-bond network with the residues Pro236 and Lys103 by a water molecule (W936), the screened compounds also formed hydrogen bonds with the key residues Lys101 and Glu138. The docking results indicated that nine screened compounds might be potential NNRTIs.

**Table 3:** Chemical structures and docking scores of the screened hit compounds as novel HIV-1 NNRTIs from ZINC database.

**Compound No**.	**Structure**	**Docking score**
ZINC_57841658	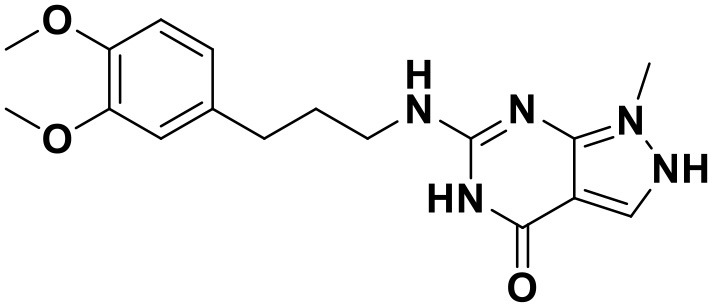	9.43
ZINC_60381334	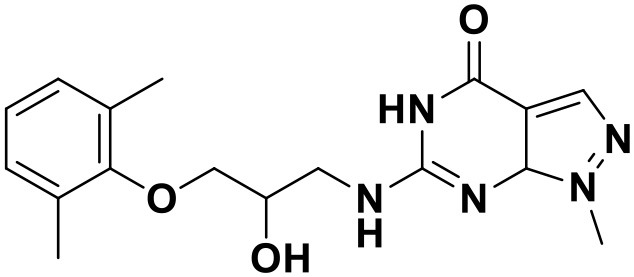	9.02
ZINC_63070905	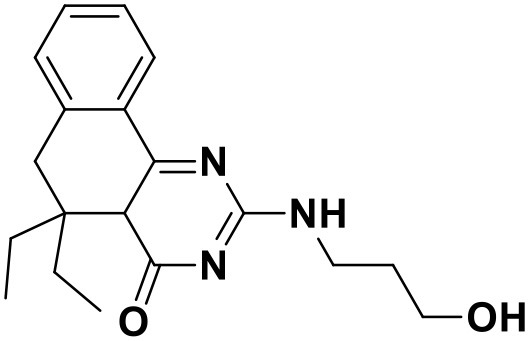	9.56
ZINC_69532225	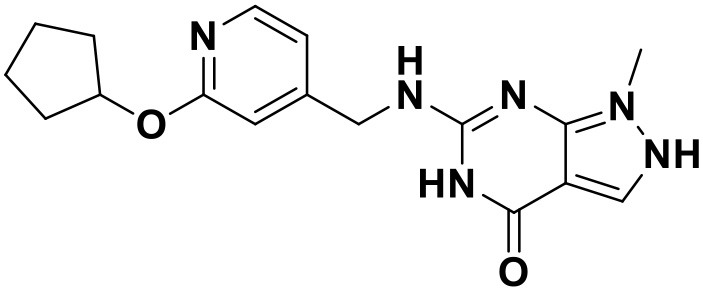	9.12
ZINC_71894576	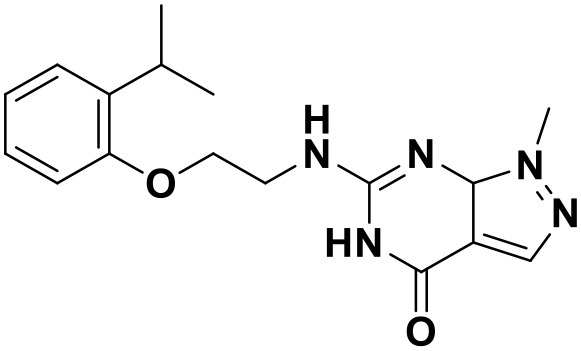	9.00
ZINC_73709240	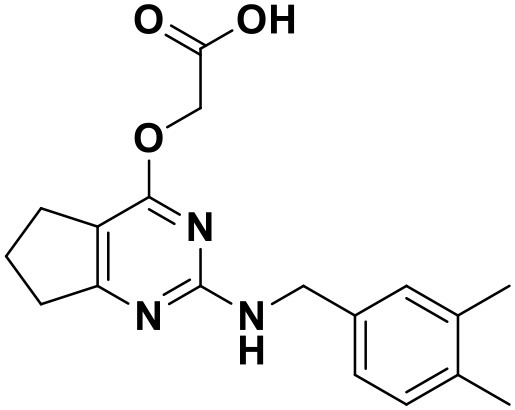	9.64
ZINC_89506228	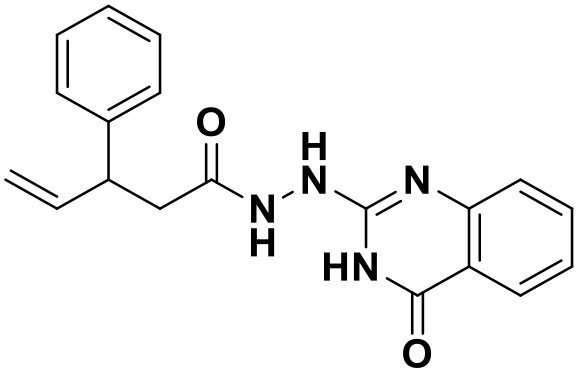	9.29
ZINC_91409938	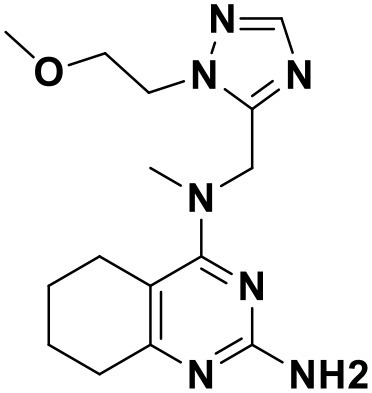	9.30
ZINC_97995063	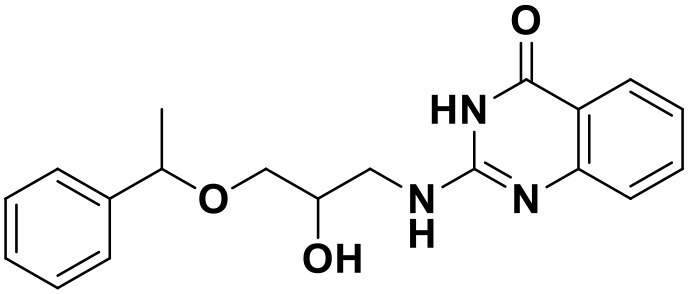	9.22

*NNRTIs, non-nucleoside reverse transcriptase inhibitors*.

### Newly Designed Non-nucleoside Reverse Transcriptase Inhibitors

According to the structural characteristics of DHPYs and the results of the 3D-QSAR models and molecular docking, we further designed three new compounds (**N1**, **N2**, and **N3**; Table 4) using ZINC_73709240 as a lead compound. The 3D-QSAR contour maps indicated that the hydrogen-bond acceptor at the *para-position* of the benzene ring of Tolerant Region II and the hydrogen-bond donor at the terminal of Tolerant Region II were favorable to the inhibitory activity. Therefore, we designed compounds **N1**, **N2**, and **N3** by adding amide or carboxyl groups as hydrogen-bond donors or acceptors at these positions (Table 4).

**Table 4:** Chemical structures and docking scores of the newly designed HIV-1 NNRTIs.

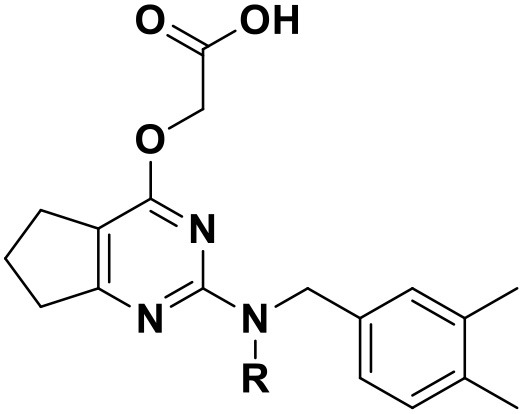			
**Compound No**.	***R***	**Docking score**	
		**Wild-type HIV-1 RT**	**Mutant HIV-1 RT (K103N+Y181C)**
N1	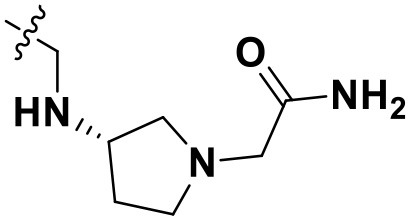	13.83	10.60
N2	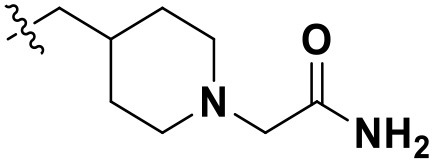	12.59	12.03
N3	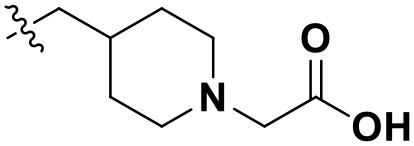	12.93	12.88

*NNRTIs, non-nucleoside reverse transcriptase inhibitors; RT, reverse transcriptase*.

**Figure 9:**
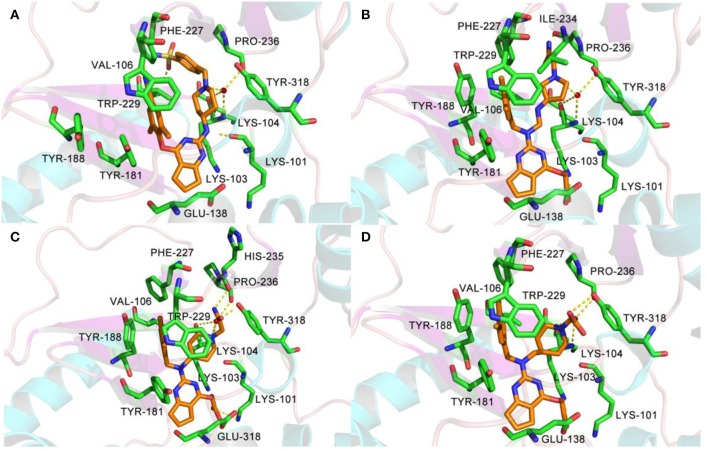
The docked results of compounds **36 (A)**, **N1 (B)**, **N2 (C)**, and **N3 (D)** in the binding pocket of wild-type HIV-1 reverse transcriptase (RT) (PDB: 6C0J).

All designed compounds were then docked into the binding site of HIV-1 RT by Surflex-Dock Geom method. The docking scores of compounds **N1**, **N2**, and **N3** were 13.83, 12.59, and 12.93, respectively, and higher than that of compound **36** (11.86), suggesting that the interactions between the newly designed compounds and the protein might be more stable. As shown in Figure 9, the binding modes of compounds **N1**, **N2**, and **N3** with the protein were basically similar to that of compound **36**. The left wings of four ligands were all located in the hydrophobic region and formed π-π stacking interactions with residues Tyr181, Tyr188, Trp229, and Phe227, and the positive nitrogen of their right wing formed hydrogen-bond networks with Lys103 and Pro236 through a water molecule (W936), which was in good agreement with the docking results. However, there were some differences for four compounds in terms of protein–ligand interactions. As for compound **36**, it formed three hydrogen bonds with Lys101 (Lys101-O…H-N-, 2.8 Å) and Val106 (Val106-NH…O=C, 3.0 Å; -O…H-N-, 2.7 Å), which was consistent with the redocked results of **K-5a2**. As can be seen from Figures 9A,B, compounds **N1** and **N3** not only formed hydrogen bonds with Glu138 and Lys103 but also had hydrogen bonds with Ile234 and/or Tyr318. Another finding was that five hydrogen bonds were formed between residues Glu138, Lys103, Lys101, Tyr318, and His235 with compound **N2**. The docking results revealed that the four compounds interacted with key amino acid residues (Lys101 and Glu138), and several new hydrogen bonds between three newly designed compounds and residues Lys103, Ile234, Tyr318, and His235 were found. These results suggested that compounds **N1**, **N2**, and **N3** might be the potential inhibitors with improving anti-HIV-1 activities.

To further explore whether the newly designed compounds could inhibit mutant HIV-1 RT, they were also docked into the mutant (K103N+Y181C) RT (PDB ID: 6C0R) (Figure S1; Table 4). The co-crystallizing ligand (**25a**) of 6C0R as a reference compound was also redocked into the binding site as displayed in Figure S1A. Kang et al. (2017) reported that the inhibitory activity of compound **25a** (EC_50_ = 5.5 ± 0.81 nM) against the K103N+Y181C mutant RT was better than that of RPV (EC_50_ = 11 ± 1.9 nM). Our docking results indicated that compound **25a** formed four hydrogen bonds with residues Lys101, Val106, Lys104, and Tyr188, respectively, and the π-π stacking and/or hydrophobic interactions were also found with residues Trp229, Phe227, and Tyr188. In addition, the residue Tyr183 played an important role in the binding site of the mutant RT and could offset the loss of π-stacking and hydrophobic interactions between inhibitors and residue Tyr181 as it was mutated to Cys181 (Das et al., 2008).

The docking scores of three hit compounds were relatively high (Table 4), especially compounds **N2** and **N3**, whose docking scores were higher than **25a** (12.02), indicating that the newly designed molecules might have better inhibitory activity against the mutant RT. It was observed that the hydrogen bond with residue Lys101, π-π stacking, and hydrophobic interactions still existed for three complexes (Figure S1). However, the difference was that compounds **N1**, **N2**, and **N3** could form a direct hydrogen bond with the mutated residue Asn103, which indicated that these designed molecules could bind well in the binding pocket with mutations. These docking results demonstrated that the three hit compounds might have the ability to inhibit the HIV-1 RT mutant. However, the actual anti-HIV activities of the three hits are necessary to be identified in future studies.

### ADME Analysis

ADME prediction studies were carried out for compound **36** and three newly designed NNRTIs (**N1**, **N2**, and **N3**). The results were depicted in Table 5. In this program, five inhibitors of cytochrome P450 (CYP) enzymes were predicted. CYPs, which primarily mediated oxidation of various compounds and participated in physiological and pathophysiological processes, were the major phase I drug-metabolizing enzymes and responsible for metabolism of about 75% of all marketed drugs (Moroy et al., 2012). In the family of CYP enzymes, the CYP3A4 was the most important enzyme on account of metabolizing ~50% of all drugs by itself, and the CYP2C9 enzyme mainly metabolizes several clinically used drugs such as celecoxib and diclofenac (Daly et al., 2017). As shown in Table 5, compounds **N1**, **N2**, and **N3** could be easier to be metabolized compared with compound **36**. In addition, three newly designed compounds showed high human gastrointestinal absorption (HIA), indicating that they might have a high chance of brain penetration (Li et al., 2019). The topological polar surface area (TPSA) values of compound **N1** and **N3** were in the range from 20 to 130 Å^2^, which suggested that they possessed good transport properties *in vivo*. Notably, the synthetic accessibilities of designed compounds were lower than 5, suggesting that they were relatively easy to be synthesized. On the whole, the ADME properties of the three newly designed compounds were superior to that of compound **36**, especially in pharmacokinetics, druglikeness, and medicinal chemistry properties. Thus, the three newly designed compounds might be supposed to have good pharmacokinetics properties.

**Table 5:** Predicted absorption-distribution-metabolism- excretion (ADME) parameters and drug-like properties of compound **36** and the newly designed inhibitors (**N1–3**).

**Properties**	**Parameters**	**Compounds**			
		**36**	**N1**	**N2**	**N3**
Physicochemical	MW^a^ (g/mol)	532.66	482.58	481.59	482.57
Properties	Rotatable bonds	7	11	10	10
	HBA	8	8	7	8
	HBD	2	3	2	2
	TPSA^b^	142.61	133.91	121.88	116.09
Lipophilicity	iLOGP	3.82	3.29	3.38	3.60
	XLOGP3	4.17	0.04	0.92	1.57
	WLOGP	4.33	0.63	1.72	2.32
	MLOGP	2.06	0.44	1.42	1.82
	Silicos IT logP	3.51	2.08	3.09	3.33
	Consensus logP	3.58	1.30	2.11	2.53
Water Solubility	ESOL Class	MS^c^	S^d^	S	S
	Ali Class	PS^e^	S	S	S
	Silicos IT Class	PS	MS	MS	MS
Pharmacokinetics	GI^f^ absorption	low	high	high	high
	BBB^g^ permeat	No	No	No	No
	CYP1A2 inhibitor	No	No	No	No
	CYP2C19 inhibitor	Yes	No	No	No
	CYP2C9 inhibitor	Yes	No	No	No
	CYP2D6 inhibitor	Yes	No	Yes	Yes
	CYP3A4 inhibitor	Yes	No	Yes	Yes
Druglikeness	Lipinski violations	1	0	0	0
	Ghose violations	2	2	2	2
	Egan violations	1	1	0	0
	Muegge violations	1	1	0	0
	Bioavailability Score	0.55	0.55	0.55	0.56
Medicinal Chemistry	PAINS^h^ alerts	0	0	0	0
	Brenk alerts	0	1	0	0
	Leadlikeness violations	2	2	1	2
	Synthetic accessibility	4.68	4.68	4.12	4.10

a *Molecular weight*.

b *Total polar surface area*.

c *Moderately soluble*.

d *Soluble*.

e *Poorly soluble*.

f *Gastrointestinal*.

g *Blood–brain barrier*.

h *Pan assay interference compounds*.

### Molecular Dynamics Simulation

**Figure 10:**
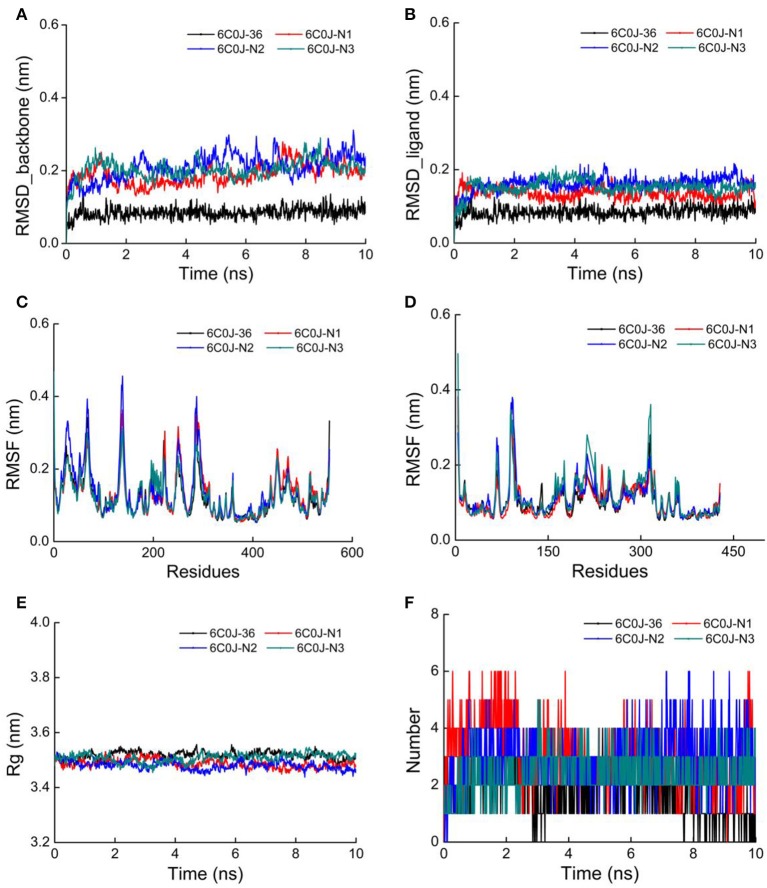
The 10 ns molecular dynamics (MD) results of compounds **36**, **N1**, **N2**, and **N3** in wild-type HIV-1 reverse transcriptase (RT). **(A)** Root mean square deviation (RMSD) values of backbone atoms of the protein. **(B)** RMSD values of the ligands. **(C)** Root mean square fluctuation (RMSF) values of the chain A. **(D)** RMSF values of the chain B. **(E)** Radius of gyration (Rg) values of backbone atoms. **(F)** The total number of hydrogen bonds between the ligands and the protein.

As for newly designed molecules, their stability of protein–ligand interactions should be taken into account. Thus, 10 ns MD simulations were performed for four complex systems, 6C0J-36, 6C0J-N1, 6C0J-N2, and 6C0J-N3, respectively. The RMSD values of backbone atoms for the four complexes were displayed in Figure 10A. During the 10 ns MD simulations, the RMSD values of the four systems were relatively stable and were lower than 0.3 nm. Figure 10B showed the RMSD values of the four ligands during 10 ns MD simulations. The four ligands had similar fluctuations and reached equilibrium at approximately 0.5 ns. The root mean square fluctuation (RMSF) profiles of the four complexes (Figures 10C,D) also exhibited similar trends during the MD simulations. It should be pointed out that the key residues, Lys101 of chain A and Glu138 of chain B, had relatively lower RMSF values. As shown in Figure 10E, the radius of gyration (Rg) values, which could explain the compactness of the protein throughout simulation, basically maintained at about 3.5 nm, indicated that greater changes of the conformations of protein did not take place. In addition, the intermolecular hydrogen bonds could be used to analyze the protein–ligand interaction. As shown in Figure 10F, the hydrogen-bond numbers of 6C0J-36, 6C0J-N1, 6C0J-N2, and 6C0J-N3 complexes were 1-2, 1-6, 1-6, and 2-5 over the 10 ns simulations, respectively, which suggested that all newly designed compounds might be more stable than compound **36**. The MD simulation results revealed that four protein–ligand complexes could maintain a relative stability in the dynamic simulation and three newly designed compounds might have more interactions with the HIV-1 RT than compound **36**. These were in good consistency with the docking results.

In the same pattern, 10 ns MD simulations were also carried out for three protein–ligand complexes (6C0R-N1, 6C0R-N2, and 6C0R-N3) to further study whether they still could remain stable in the dynamic environment. The results were shown in Figure S2. The RMSD values of protein backbones of the three complexes were displayed in Figure S2A, and it can be clearly seen that they basically reached stability after 5 ns and were below 0.4 nm. The RMSD values of the three ligands were also stable (Figure S2B). Figures S2C,D were the RMSF plots of chains A and B, respectively, which showed that the residues of the three complexes fluctuated in the same trend, indicating that they had great stability. In addition, the Rg values just slightly floated within 3.5 nm from Figure S2E, indicating that the proteins had good compactness. The number of hydrogen bonds was also essential to verify the stability. As shown in Figure S2F, the hydrogen-bond numbers of compounds **N1**, **N2**, and **N3** were 2-4, 1-5, and 2-5 over the 10 ns MD, respectively, suggesting that the three compounds could tightly bind to the mutant RT. The abovementioned results revealed that the three complexes could keep stable during MD simulations and the three designed compounds could interact well with the mutant HIV-1 RT. However, the experimental activities of the three new hits against wild-type and mutant HIV-1 strains remain to be studied.

## Conclusion

In conclusion, 52 DHPYs were collected to construct the CoMFA and CoMSIA models, which exhibited rationally statistical parameters and good predictive ability. These models well-explained the 3D-QSARs of these DHPY and provided useful information for designing new HIV-1 NNRTIs. The optimal pharmacophore model containing eight features was in agreement with the 3D-QSAR results. The docking results revealed that Lys101 was the key amino acid residue, and the hydrophobic and π-π stacking interactions with Tyr181, Tyr188, Trp229, and Phe227 also played key roles for the anti-HIV activity of DHPYs. Nine lead compounds were obtained by the pharmacophore-based and docking-based virtual screening as well as ADME prediction. Three novel inhibitors were designed by modifying the structure of the screened compound ZINC_73709240 according to the 3D-QSAR and docking results. Three newly designed inhibitors showed good stability and strong interactions not only in the wild-type RT but also in the K103N/Y181C RT mutant based on the docking and MD simulation results. The ADME prediction indicated that compounds **N1**, **N2**, and **N3** might possess desirable drug-like properties. However, further study on synthesis and anti-HIV activities of the three newly designed hits is necessary. We expect that the screened and designed compounds could be served as lead candidates of novel HIV-1 NNRTIs.

## Data Availability Statement

All datasets generated for this study are included in the article/Supplementary Material.

## Author Contributions

GL and YC proposed the research idea and designed the experiment. YC performed the experiment. YC, FW, YT, and YG analyzed the data. YC, XL, XJ, and GL wrote the manuscript. All authors revised and approved the manuscript.

### Conflict of Interest

The authors declare that the research was conducted in the absence of any commercial or financial relationships that could be construed as a potential conflict of interest.

## Footnotes

^1^UNAIDS. *UNAIDS Data 2018*. Available online at: http://www.unaids.org/en/resources/documents/2018/unaids-data-2018
