# Supplementary material for: In silico Design of Novel HIV-1 NNRTIs Based on Combined Modeling Studies of Dihydrofuro[3,4-d]pyrimidines
Source: Front Chem. 2020 Mar 24;8:164. doi: 10.3389/fchem.2020.00164 (PMC7105726; doi:10.3389/fchem.2020.00164)
Supplement: Supplementary file 1 [file Presentation_1.pdf]

Supplementary Material

In silico design of novel HIV-1 NNRTIs based on combined modeling studies of dihydrofuro[3,4-d]pyrimidines

Yanming Chen [1], Yafeng Tian [1], Ya Gao [1], Fengshou Wu [1], Xiaogang Luo [1,2], Xiulian Ju [1], Genyan Liu [1,*]

[1] *Key Laboratory for Green Chemical Process of Ministry of Education, Hubei Key Laboratory of Novel Reactor and Green Chemical Technology, School of Chemical Engineering and Pharmacy, Wuhan Institute of Technology, Wuhan 430205, P. R. China*

[2] *School of Materials Science and Engineering, Zhengzhou University, No.100 Science Avenue, Zhengzhou City, 450001, Henan Province, P. R. China*

*Correspondence: Genyan Liu, School of Chemical Engineering and Pharmacy, Wuhan Institute of Technology, Wuhan 430205, Hubei, P. R. China

Tel.: +86-136 6723 5670

Email Addresses: liugenyan@yahoo.com; liugenyan@wit.edu.cn (G. Liu)

Table S1. Classical statistical parameters of the CoMFA and CoMSIA models.

	**Model**	**q^2**	**ONC**	**R^2**	**SEE**	**F**	**r_{pred}^2**	**Field contributions (%)**				
								**S**	**E**	**A**	**D**	**H**
CoMFA	**S+E**	**0.647**	**8**	**0.970**	**0.094**	**121.235**	**0.751**	**42.9**	**57.1**			
	**S+E+A+D+H**	**0.735**	**10**	**0.982**	**0.076**	**151.015**	**0.672**	**4.1**	**19.7**	**29**	**33.4**	**13.8**
	A+H	0.562	6	0.919	0.149	60.902	0.542			60.8		39.2
	S+H	0.593	7	0.916	0.154	48.294	0.432	22.1				77.9
	A+D+H	0.684	11	0.982	0.076	136.118	0.346			34.8	44.1	21.1
	E+D+H	0.711	8	0.947	0.125	66.611	0.389		34.2		21.3	44.5
CoMSIA	E+A+H	0.605	5	0.905	0.159	63.062	0.538		35.2	42.3		22.5
	S+A+H	0.632	12	0.974	0.094	80.698	0.657	12.7		48.9		38.4
	E+A+D+H	0.723	11	0.983	0.074	143.976	0.601		20.9	29.4	35.1	14.6
	S+A+D+H	0.685	11	0.983	0.074	144.841	0.379	5.1		34.1	41.5	19.3
	S+E+D+H	0.705	8	0.947	0.125	66.748	0.453	4.9	32.1		43.2	19.8
	S+E+A+H	0.634	5	0.913	0.152	69.200	0.616	46.7	32.4	39.8		21.2

Table S2. Statistical parameters of the pharmacophore models.

Name	SPECIFITY	N_HITS	FEATS	PARETO	ENERGY	STERICS	HBOND	MOL_QRY
Model_01	5.346	7	10	0	268.32	5458.10	601.20	156.74
Model_02	2.914	10	10	0	19056660	5442.80	700.90	186.64
Model_03	5.785	10	8	0	655334.44	5379.40	636.50	207.01
Model_04	4.231	10	9	0	1814494.5	5292.30	667.70	142.97
Model_05	5.334	10	10	0	2932319	5707.10	602.80	129.35
Model_06	4.423	7	8	0	247.97	4924.60	595.30	124.69
Model_07	5.302	7	9	0	1831234.8	5550.00	612.90	99.63
Model_08	5.413	7	9	0	332.48	4682.30	602.30	156.71
Model_09	5.400	10	9	0	261107.9	4982.00	656.90	93.39
Model_10	4.550	9	8	0	10088992	5325.90	667.20	104.94
Model_11	4.693	10	8	0	457813.1	5434.80	617.10	92.35
Model_12	5.443	7	9	0	1421060	5237.60	676.30	84.12
Model_13	5.576	10	8	0	18232012	5511.30	659.90	81.53
Model_14	5.658	7	8	0	5499636	5733.70	653.50	76.31
Model_15	4.537	7	9	0	355631.6	4983.10	619.90	92.48
Model_16	4.498	5	10	0	1718275.7	5461.00	558.50	121.01
Model_17	5.673	10	8	0	1843.74	5338.30	608.50	65.64
Model_18	4.498	7	8	0	496847.31	5149.30	646.80	66.92
Model_19	4.554	8	8	0	37.30	4663.60	526.90	85.87
**Model_20***	**5.746**	**9**	**8**	**0**	**62.29**	**4521.80**	**568.20**	**70.88**

Table S3. Hydrophobic, hydrogen bond, and π-π stacking interactions of the screened hits as potential HIV-1 NNRTI in the RT protein (PDB ID:6C0J).

Compounds	Hydrophobic	Hydrogen bonds	π-π
ZINC_57841658	Val106, Tyr181, Tyr188, Phe227, Trp229	Lys101 with O of NH<br>Lys101 with OH of N<br>Lys101 with NH of C=O	Tyr188, Tyr181, Phe227, Trp229
ZINC_60381334	Val106, Tyr181, Tyr188, Phe227, Trp229	Lys101 with O of NH<br>Lys101 with OH of N	Tyr188, Tyr181, Phe227, Trp229
ZINC_63070905	Val106, Tyr181, Tyr188, Phe227, Trp229	Lys101 with O of NH<br>Glu138 with OH of OH	Trp229
ZINC_69532225	Val106, Tyr181, Tyr188, Phe227, Trp229	Lys101 with O of NH<br>Lys101 with OH of N<br>Lys101 with NH of C=O	Tyr188, Tyr181, Phe227, Trp229
ZINC_71894576	Val106, Tyr181, Tyr188, Phe227, Trp229	Lys101 with O of NH<br>Lys101 with OH of N<br>Lys101 with NH of C=O	Tyr188, Tyr181, Phe227
ZINC_73709240	Val106, Tyr181, Tyr188, Phe227, Trp229	Ile180 with OH of C=O<br>Ile180 with NH of C=O<br>Glu138 with OH of C=O	Tyr188, Tyr181, Phe227, Trp229
ZINC_89506228	Val106, Tyr181, Tyr188, Phe227, Trp229	Lys101 with O of NH<br>Lys101 with OH of N<br>Lys101 with NH of C=O	Tyr188, Tyr181, Phe227, Trp229
ZINC_91409938	Val106, Tyr181, Tyr188, Phe227, Trp229	A network between Pro236 and Lys103 by a water molecule (W936)	Tyr188, Tyr181, Phe227, Trp229
ZINC_97995063	Val106, Tyr181, Tyr188, Phe227, Trp229	Lys101 with O of NH<br>Lys101 with OH of N<br>Lys101 with NH of C=O	Tyr188, Tyr181, Phe227, Trp229

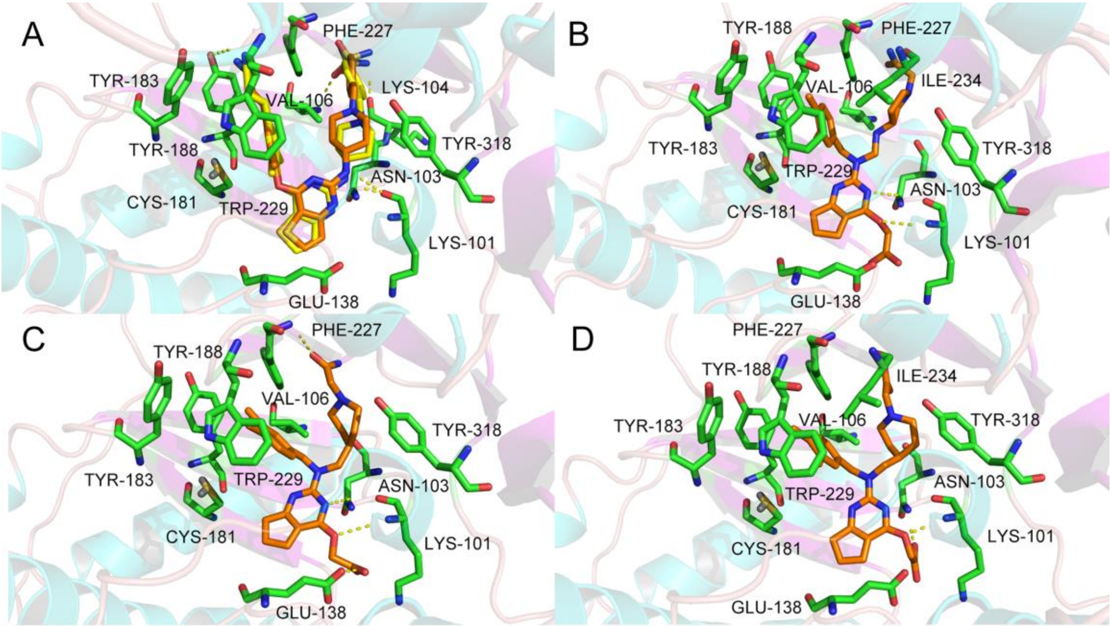

Figure S1. The docked results of compounds **25a**, **N1**, **N2**, and **N3** in the binding pocket of the mutant HIV-1 RT (PDB ID: 6C0R). (**A**) The re-docked co-crystallizing ligand (**25a**). The original and re-docked conformation were colored in yellow and orange, respectively. (**B**) The docking mode of compound **N1**. (**C**) The docking mode of compound **N2**. (**D**) The docking mode of compound **N3**.

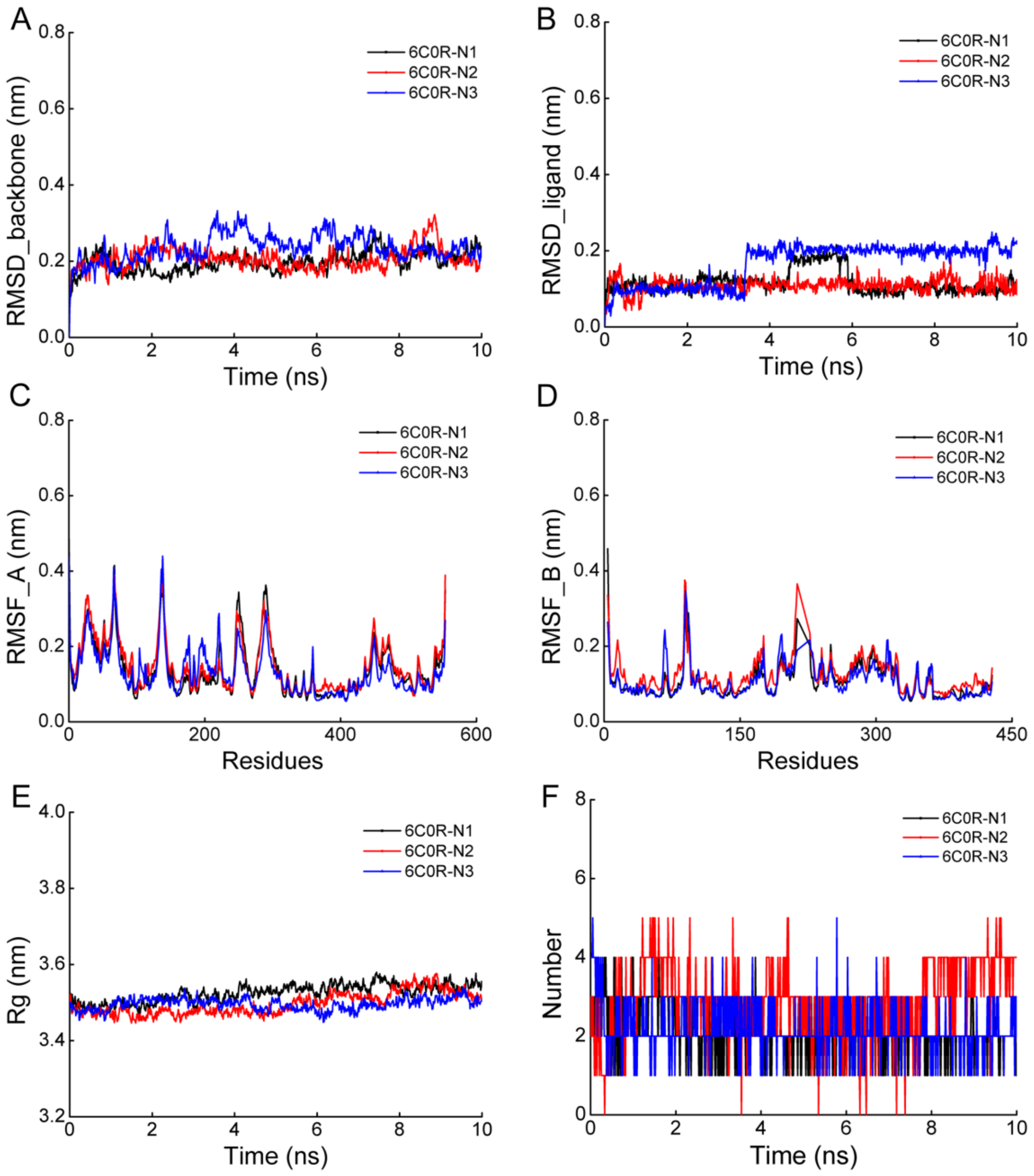

Figure S2. The 10 ns MD results of compounds **N1**, **N2**, and **N3** in the mutant HIV-1 RT (PDB ID: 6C0R). (**A**) RMSD values of backbone atoms of the protein. (**B**) RMSD values of the ligands. (**C**) RMSF values of the chain A. (**D**) RMSF values of the chain B. (**E**) Rg values of backbone atoms. (**F**) The total number of hydrogen bonds between the ligands and the protein.
